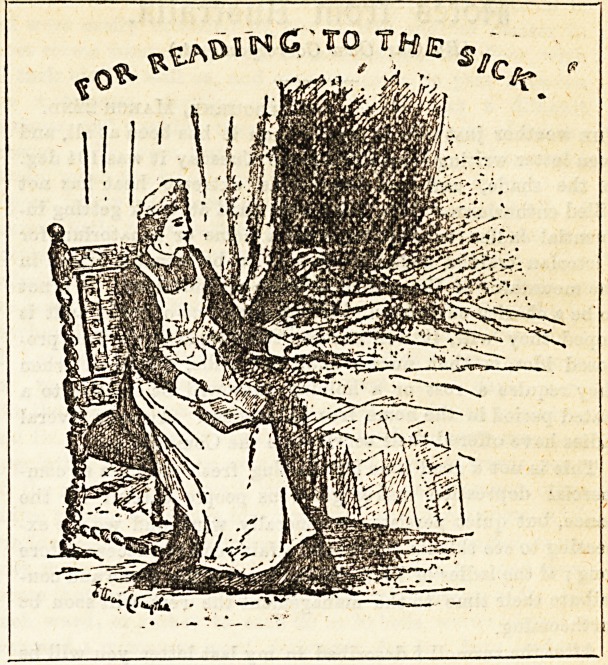# The Hospital Nursing Supplement

**Published:** 1892-05-14

**Authors:** 


"The Hospital" Nursing Mirror.

Being the Extra Nursing Supplement of "The Hospital" Newspaper.

Contributions for this Supplement should be addressed to the Editor, The Hospital, 140, Strand, London, W.C., and should have the word "Nursing" plainly written in left-hand top corner of the envelope.

### En Passant.

ST. GEORGE'S HOSPITAL NURSES. — Accommodation for twenty-five nurses is being made in Montpellier Street, Brompton Road; the houses are being put into thorough repair and made suitable for the purpose.

THE L. O. S. EXAMINATION.—In April last the five pupils sent up from St. John's Maternity Home, Battersea, successfully passed the London Obstetrical Society's examination. The names were: Miss Alice Brickwell, Mrs. Earle, Nurse Fitchett, Nurse Goodman, and Nurse Maiben.

THE STOCKTON AND DISTRICT NURSING ASSOCIATION.—The year's income was £778, while the expenditure was only £185 17s. In most cases the reverse order of things is the rule. Stockton has an excellent system of subscription for the Nursing Fund; sixteen works in the town contribute systematically 1d. per man per month; we should like to see other towns support the nursing in the way the Stockton men do. They have our hearty congratulations on the success of their first year's work.

THE WELSH BRANCH OF THE Q.V.J.I.N.—We are glad to see that, in spite of some uphill work, this branch, with Miss Heath as Lady Superintendent, is gaining ground, and as the increase of work over the first year has been just doubled the need for the workers among the sick poor cannot be doubted for one moment, witness the fact that 942 visits were paid in December, 1890, as against 1,647 in December, 1891. Miss Kate Jones has gone to work at Conway, and Miss Evans at Barry, and three new Welsh-speaking probationers have joined the Home. The year began with a balance of £189, which will not go far towards meeting what must necessarily be increased annual expenditure. We congratulate whoever is responsible for the bringing out of this report; it is most clear and business-like, and has the merit which we commend to other institutions, that anyone glancing at it can gain a complete knowledge of the work and scope of the Society, whereas there are many reports which are as Hebrew to the uninitiated, owing to the fact that the writer of the report knows all about his institution, and takes it for granted that others are in a like position.

COOKERY.—From Bonthron's Diabetic Bread to Benoist's dainty French pastry may seem a long flight to take, but it is one easy of accomplishment at the sixth annual exhibition of the Universal Cookery and Food Association, which was opened by the Lord Mayor on May 3rd. He said, in the course of a speech which dealt exhaustively with the question of food, that he hoped this would be as successful as those previous exhibitions from which the proceeds, amounting to about £1,300, had been handed to various London hospitals—a hope cordially concurred in by the audience. That food, whether of the simplest or the most expensive sort, can be made thus attractive to look at, as well as appetising and wholesome, was clearly evinced by the infinite variety displayed in the Portman Rooms. We saw a preparation of eucalyptus in the form of "sweets," which is said to be a palatable form in which to take this valuable drug when "a heavy cold" threatens us.

NURSES FOR INDIA.—We are glad to say that several well-known Anglo-Indian ladies have already come forward in answer to Mrs. Cuthell's letters, and have volunteered their help in the promotion of a scheme, which we hope ere long to see thoroughly matured, to supply India with sick nurses other than military. We shall be glad to hear of anybody willing to further Mrs. Cuthell's plan.

SHORT ITEMS.—Miss E. A. Baker, of South Africa, and Miss Susan G. Dougall, of Montreal, have passed the final examinations of the Royal College of Physicians and Surgeons of Edinburgh, and of the Faculty of Physicians and Surgeons, Glasgow, and are now on the register as duly qualified medical practitioners.—Some of the women students at the Medical School in Moscow have volunteered and gone to the Samara district to nurse the victims of a bad outbreak of typhoid.—Mrs. Selfe Leonard writes an article in this month's "Biblewomen and Nurses" weighing the merits of the woman of the people as a district nurse against the lady in the same capacity. One of the points in favour of the former is that two of Mrs. Leonard's nurses can be kept for what one "lady" nurse costs to keep.—A Lady Superintendent writes to us to testify to the efficiency of the masseuses who have received their training from Mrs. C. Hales.

NURSES AT COLNEY HATCH.—It is with great pleasure that we hear that the nurses at this asylum, who went in for the "first aid" examination last year, have all now been up successfully for the "nursing" examination. This time, however, fourteen nurses only were up, as against twenty for the "first aid" examination, but this is owing to the nurses having left to be married, and for one reason or another, not for any lack of zeal in wishing to further their training knowledge.

The "written" examination questions will interest many of our readers. The examiner was Dr. H. P. Potter.

1. Enumerate the infectious fevers. Give the history of one, say scarlet fever.
2. How do you prepare the bed for a compound fracture of the thigh?
3. In what way may wounds heal; and what are the various antiseptics used for wounds?
4. What is the temperature of a hot bath? What precautions are taken in giving hot baths?
5. What is meant by ventilation; and by what natural forces is the air changed in the apartment?

The Superintendent, the Matron, and Mr. C. T. Ewart, who prepared the class, all realise the importance and necessity of the training of attendants, and it is refreshing to find that the Committee were most willing to help with the necessary funds for these examinations. Is it too much to hope that the ball has really been set rolling, and that little by little the great fact that unskilled nursing is a hindrance in the treatment of our insane is forcing its way? At all events, such facts as these we publish to-day are cheering "stubborn things," and the teachers and the nurses are helping to bring nearer that eventful day when a Medical Superintendent will be unworthy of his name if he fails to give his nurses all the instruction in his power. The following are the names of the successful candidates: Nurse Ashfield, Nurse Bovington, Nurse Baker, Nurse Barnes, Nurse Cotterell, Nurse Wear, Nurse Ellis, Nurse Gosborne, Nurse Greenaway, Nurse Kentsley, Nurse Mingay, Nurse Nelson, Nurse Welch, Nurse Wise.

### Ventilation, Disinfection, and Diet.

By P. Caldwell Smith, M.D.

### V.—TEMPERATURE OF SICK ROOMS—VENTILATION OF HOSPITALS—VENTILATION OF SICK ROOMS.

I have now tried to explain with as little technical detail as possible the main principles of ventilation, and we are now in a position to ask ourselves the question, What is the best method for the ventilation (1) of hospitals, and (2) of sick rooms in an ordinary house? Before proceeding to give an answer to this question, let me first make a few remarks regarding the temperature at which rooms should be maintained.

It is, of course, quite customary nowadays to have in each sick room or hospital ward a thermometer, that the temperature may be kept as equal as possible, but I would prefer that, instead of an ordinary thermometer, a wet and dry bulb thermometer should be used, so that not only the absolute temperature could be ascertained, but also the amount of moisture present, or what is called the humidity. The wet and dry bulb thermometers are easily read, and by reference to a table the amount of humidity is at once got. The amount of humidity should not be greater than 75 per cent., but it may be less without producing any ill effect if the supply of fresh air be good. The dry bulb thermometer should not read less than 60 deg., nor more than 65 deg., although in some diseases, as, for example, typhus fever, where free ventilation is a necessity, a lower temperature may be quite safe.

The wet bulb thermometer ought to read 57 deg. F. to 61 deg. F. To put it more easily, the difference between the wet and dry bulb thermometers should not be less than 4 deg. F., nor more than 8 deg. F. In wet weather the air is what is called saturated, or full of moisture, and consequently outside the two thermometers read the same; but in all inhabited rooms, and especially in sick rooms and wards of hospitals, this should never be allowed to occur. To prevent this from happening, we have only to heat the room sufficiently, and this dries the atmosphere, so that the wet bulb will read lower. For example, if the air outside be at 50 deg., and saturated as in wet weather, by warming the room to 63 deg. F., the air entering will never produce an excess of moisture in the apartment.

Hospitals must be ventilated according to their size and the nature of the diseases treated in them. For example, in a small cottage hospital, say of 20 beds, with four or six beds in each ward, and of one storey, natural ventilation will be found quite satisfactory. Hot water coils placed between each window, and the inlets for fresh air being also placed there, so that the air is heated by these coils, with a good Buchan ventilator on the roof, will be found quite satisfactory. Of course, it may be necessary to have also an open fireplace, and I would prefer for this a Galton ventilating stove, so that heated air is also introduced. The windows should also be made to open as described in Hinckes Birds system, so that in summer additional air could, if necessary, be introduced. This same system is also applicable to fever hospitals designed on the separate pavilion system, as at Belvedere Fever Hospital, Glasgow. When the air is stagnant the Buchan ventilator will not act well, and to remedy this, Bunsen burners should be placed in it, so that, by heating the air, an upward current is produced and the extraction of foul air from the ward carried on.

In all large hospitals it will be found necessary to use some mechanical means for ventilation, and this is specially the case if the hospital be more than one storey. As already stated, this may be effected either by propelling or extracting fans; but the best authorities differ as to which of these is the better method. I am inclined to think that extraction is the best, although I am aware that the New Victoria Infirmary is ventilated on the propulsion system, and that it is said to work satisfactorily. One objection to the system as carried out there is that the foul air is drawn off near the floor of the wards instead of at the top; but further experience of the system there will be necessary before pronouncing as to its efficacy.

Mr. Douglas Galton does not believe in the propulsion system except for hot climates, the air being cooled in underground channels before passing into the rooms or wards. Lately, he has spoken very highly of Mr. Key's method of ventilating the Victoria Infirmary by the propulsion system. In the ventilation of Hillhead Public School, Glasgow, an extracting fan is used, and of course wards could be ventilated in the same manner. The fresh air is warmed in winter or cooled in summer in the basement, and flows into the room at proper inlets, while the foul air is extracted by outlets carried by means of tubes towards the fan placed at the roof. In addition to these outlets there ought also in hospitals to be outlets near the floor which could be used in cold weather, drawing off the cold air near the floor and causing the warmer air nearer the ceiling to circulate through the ward. I would decidedly recommend that whatever system of ventilation be used open fireplaces should not be abolished, and for this reason: in mechanical systems of ventilation, where the air is warmed before admission, the walls of the room are only heated to a very small degree. The great reason of the comfort of the open fire is that it warms the walls first, and the air of the room is warmed by the walls. In heating by hot water or steam-pipes some portion of their heat is radiated to the walls, the high-pressure system of heating being the more effective. Consequently, open fireplaces are a necessity, and should always form part of any system of heating and ventilation.

We have now to consider perhaps the most difficult problem of all, viz., the ventilation of a sick room in an ordinary house, with no special means provided. It is always necessary to remember that you must have an outlet for foul air and an inlet for fresh air. In ordinary circumstances the only outlet is the open fire, and this should always be kept burning. In summer even, although the temperature be above 70 deg., yet the fire should be kept on, perhaps just sufficient to have the chimney heated and an up draught caused. If this causes the room to be overheated, then a lamp can easily be placed in the grate, which will heat the chimney sufficiently to cause it to act as an outlet for foul air.

In summer the windows may be more freely used without fear of producing draughts, but the fresh air should never be introduced low down, that is, at the foot of the lower sash. I prefer instead of lifting the lower half of the window, and inserting a board there, to draw down the upper half for three or four inches. Then get a piece of wire gauze or perforated zinc, the length of the window space, and six inches broad, and nail it across the top. This effectually breaks up the air currents, and, as is often the case when it is an inlet, prevents draughts blowing down on to the bed, or the heads of the occupants of the room. The space between the sashes, owing to its construction, allows of the current being directed upwards towards the ceiling. For inlets, the window may also be used as above described, but if this cools the room too rapidly, as ascertained by the thermometer, then the door can be used as an inlet. This may be done as follows: A screen is procured exactly of the same size as the door. The door is half opened, and the screen is placed so as to form another door, but hinged on the opposite side. Thus two doors are formed, and the air flows into the room through the triangular aperture at the top. This is specially useful if the door opens into a hall or corridor heated by hot-water pipes, as is often the case.

This as an inlet, with a good drawing fire as an outlet, I have found to be a fairly satisfactory solution of a somewhat difficult problem, and I hope it, as well as some of the foregoing, may prove useful to you all in the treatment of disease.

### The Metropolitan and National Nursing Association.

A large gathering of the friends of this Association took place on Monday afternoon at Grosvenor House, when the Duke of Westminster opened the proceedings, and presented the sixteenth annual report. He spoke of it as a quietly working institution, happy in the possession of "no history," and proceed to explain the objects of the Association, as set forth in the report.

The next speaker, Mr. Mocatta also referred to the increasing demand for qualified nurses, to be trained on the lines so successfully followed by the M. N. N. Association.

Sir Henry Acland made a most interesting speech and referred to his own experience, gained during a long life, comprehending the days of the Crimean War, "when Miss Nightingale taught us what we might expect." He had himself gone on her rounds with a district nurse in both St. Giles and East London and he was amazed at what he saw, and the skill and knowledge which the cultivated ladies brought to bear on every detail in the poorest of homes. These nurses not only produce a certain beneficial effect on the sick person but often have a moral influence on the whole house, and Sir Henry Acland mentioned a case where a whole alley was benefitted by the example of cleanliness, neatness, and method.

Dr. Cheadle spoke of the Association as doing a good work, with no creed distinctions and no proselytising, and no humiliation of almsgiving. He had known the Association from its infancy, and when he was one of the physicians at the Great Ormond Street Children's Hospital his cases had received valuable aid from these nurses. He referred to the valuable assistance rendered by intelligent nurses to doctors, and spoke of the good work specially connected with the infectious diseases, where the discovery, management, and notification thereof was frequently done entirely through her. Almost greater was the value of the indirect results of nurses' work—the practical lessons in hygiene, the moral influence, &c.

Mr. Boyd Carpenter dwelt at some length and with great cordiality on the work of the Association.

Mr. Bousfield spoke of the pioneer society, as he called the Association, demanding followers and imitators elsewhere and everywhere. He regretted that the work done by her nine daughters (the branch societies) was not embodied in the report. He also spoke of the number of poor people who could not be taken into infirmaries or hospitals, but who must stay at home with no power to pay for skilled nursing, and he considered this last "a necessity of life" in sickness. There are twenty institutions in the country managed by local committees, but every new institution in the country demanded to have nurses supplied to it by the Bloomsbury centre, and this was beyond its power. A letter from Miss Grey, of the Clapham branch, spoke of the co-operation of the Invalid Children's Aid Association with regard to the loans of spinal carriages and other comforts for sick children. Very useful work had been done in visiting the Board Schools and attending to small but painful ailments, such as broken chilblains, sore fingers, &c., also valuable information afforded with regard to more serious injuries which have been sent to obtain proper skilled treatment.

Miss Hughes and her nurses were cordially thanked for their enthusiastic work and loving devotion, and Mr. Dacre Craven moved a vote of thanks to the Duke of Westminster for his hospitality, and acknowledged the deep debt of gratitude due to him from many charitable associations, &c.

The ordinary expenditure of the home amounted to £1,420 13s. 8d., £7 less than in the previous year.

"THE VINEYARD."

"Go work in my vineyard" is the invitation given to all Christians by our Lord, for we were hired to Him in Baptism, and He expects us to fulfil the duties we have undertaken. Some persons recognise this, and take up their work at once. These happy souls feel the comfort and support of being "always with the Father," and sharing in the blessings He bestows on His children. But others forget their responsibility, and lead careless lives, thinking merely of themselves and their pleasures. Then Christ renews the call, at the third hour when we are young, again at the sixth, the ninth, and even the eleventh hour, when our working day seems so nearly over, that it is hardly worth while to put our hands to the plough.

But He knows best, and says, "Gather up the fragments of time, health, opportunities that remain, that nothing be lost."

Many may say, "I suppose we have been hired by Christ and are His servants, but I had forgotten it, and I am sure I cannot remember any invitation given to me personally." Perhaps not; we never can hear unless the Holy Spirit prepares our hearts, and hitherto his "still small voice" has been drowned by earthly pleasures and cares, though these latter, if we had paid attention to them, were really the means by which God was reminding us of our work. And when we would not hear or understand His whispers, He drew us on one side by sickness, and now says distinctly, "Go work in my vineyard." But we reply, "How can we work when we are racked with pain? It is impossible, utterly useless to try when we cannot move from our sick beds." Have we never heard that "They also serve, who only stand and wait"? and cannot we wait patiently and take up the duties which lie near at hand?

Our own vines want trimming, possibly; they may have run wild through neglect, so we will begin with them, and see what shoots must be first nipped off. There is the wilful one. We have loved to have our own way, and go just as we choose; now we must be content to have our wills curbed and grow in the way God chooses. If we are impatient, we will try to bear our sufferings cheerfully; or scornful in the past, we can in the present receive gratefully the attentions of those about us. Though we are nailed to a sick bed, yet, like the vine on the trellis, we shall bring forth better fruit and more plentiful. The memory that He who calls us to labour knew what it was to work in hunger, fatigue, and poverty, without a place wherein to lay His head, will give us strength to bear the heat and burden of the day.

We will work while it is day in the vineyard of our Lord, using the only implement we have at hand, but the most powerful one—prayer.

### Notes from Australia.

(*By Our Own Correspondent.*)

Melbourne, March 22nd.

The weather just now is hotter than it has been at all, and even letter writing is an effort; one Tuesday it was 104 deg. in the shade, and yet even this extreme heat has not killed enthusiasm. Mrs. Hillier is hard at work getting influential ladies to help organise a home or sanatorium for Victorian trained nurses, which is to be situated either in the mountains of Victoria or at some sea-side spot. It is not to be a charity entirely, and if the nurses co-operate, as it is hoped they will, it will become self-supporting. The proposed idea is that nurses shall subscribe, and then when they require a rest or a holiday they will be entitled to a stated period in the home free of expense. Already several ladies have offered their services on the Committee.

This is not a good time for starting fresh schemes as commercial depression makes generous people careful for the nonce, but quiet persistence generally wins, and we are expecting to see this sanatorium on a fair road to success before long; if the ladies of Victoria will furnish the home and contribute their time to the management the rest will soon be forthcoming.

After the turmoil I described in my last letter you will be glad to know that matters are quieter at the Austin Hospital; it was resolved at the fortnightly meeting that a resident Medical Superintendent should be appointed to take entire charge of that institution; also that three months' notice be given to Dr. James and Miss Morley of the intended appointment which involves the termination of their respective appointments as Local Medical Officer and Matron. A few alterations have been suggested in the hospital kitchens, and a good many other improvements have been brought forward. We are all hoping the hospital will sail in smooth waters now. Our Melbourne District Nurses continue to go their way, doing a good deal of good and winning much gratitude. Lord Hopetoun came back from his country holiday just to preside at the annual meeting, and everyone thought it exceedingly good-hearted of him. The year closed with a balance of £838 16s. 4d., and as we began the year with only £127 8s. 6d. in the Hon. Treasurer's hands, we were very much pleased. The home for the nurses, which was opened in October, has been a great convenience, especially now the scope of the work has extended, and the third nurse is at work. Lady Hopetoun, who is just now with her infant "Australian" son, has been elected Patroness of the Society.

If it were not that to anybody who really cares about hospital work these constant disagreements are known to do much harm, the present disagreement at the Ballarat Hospital would really have a comical aspect. Mr. Morrison is Resident Surgeon and Superintendent, and the Secretary has lately taken to signing himself as "Secretary and Superintendent," so Dr. Morrison wrote to the Committee to enquire when the Secretary was appointed to the post to which he himself had been appointed, and which position he was not aware of having vacated.

Much discourtesy has been shown by the Committee in sending an impertinent letter to Dr. Morrison, but the whole medical staff supported the real Superintendent. The idea of the "claimant" to the envied position is funny, if it were not a clear sign of laxity in administration.

I am sorry to say that people are so vigorously putting down expenses that even nurses are among "what cannot be afforded," and some are out of employment.

The morphia habit has been finding victims over here. Two doctors, one at St. Kilda and one at Lancefield, have died lately from overdoses of the drug. It is a disgusting habit, and the knowledge of its danger seems to be of not the slightest value in preventing it.

The "unemployed" problem is still unsolved, though Government and private philanthropy are endeavouring to do something to relieve the difficulty, and the Salvation Army has opened a labour bureau. That alarmists have exaggerated the extent of the distress has been proved by personal investigation.

This being the great snake-biting month, we are once more hearing various opinions on various cures. Dr. Mueller wrote a very exhaustive article in the *Argus* supporting the merits of the strychnine cure. He was very severe on "test cases," so-called, and censures Mr. Howard Willoughby, who has been writing against the strychnine cure, very considerably, for his belief only in experiments on animals. Dr. Mueller, like many others of us, does not believe in wholesale vivisection and dog-poisoning, regardless of any knowledge as to the physiological action of the drugs employed. Up to now, physicians are not quite fearless enough in their use of strychnine, and several cases lately succumbed to the snake poison which might have been saved, in all probability, had the strychnine been injected in sufficient quantity. On February 16th, a lady resident of Hickman's Creek was bitten by a tiger snake, her husband chopped off the bitten finger, and she was taken to Avoca, where she arrived in a state of collapse. The strychnine treatment was carried out fully, and she has returned home completely cured.

The Australian Health Society goes on its way, and the Ladies' Committee have announced a series of health lectures, to commence in April.

### Examination Questions.

The following were the questions for candidates at the Medico-Psychological Association Nursing Examination, May 2nd, 1892.

1. Describe in detail the precautions which you would adopt in a case committed to your care as suicidal.
2. Mention the indications which would lead you to report a case as probably suicidal.
3. Describe carefully the precautions to be adopted for the prevention of bedsores.
4. What is meant by the work "feverish"? What observations would you make in a case where you thought the patient was feverish.
5. A recent case is admitted to your ward, and you are directed to report thereon at the next medical visit. Describe what you would look for, and how you would proceed.
6. What varieties of cases require to be fed by an attendant? Describe the procedure in each.
7. A patient in your charge is seized with an epileptic or epileptiform fit. What do you do? What do you observe?
8. What means should an attendant adopt to check dirty and destructive habits?
9. In a case of hæmorrhage what could you do till medical aid arrived?
10. What precautions would you adopt in a case of paralysis, and what events would you look out for?
11. Describe in detail any case which has been recently under your charge, noting the physical and mental symptoms in the order of their appearance.
12. What is "the insane ear," to what is it attributable, and in what classes of cases is it most common?

### Death in Our Ranks.

We much regret to announce the death of Miss Edith Green, Matron of the International Hospital, Naples. Miss Green had not long entered on her duties, and a short illness has resulted in her death.

### Experiences of a Trained Nurse in Valparaiso during the Revolution.

IT has been suggested to me that a short account of the Ambulance which was started here after the battle of Placilla might interest some of THE HOSPITAL readers. I begin by giving a translation of a letter written by Dr. T. Von Schrœder as a preface to the report lately published. "During the week which intervened between the battles of Concon and Placilla, August 20th to 28th, 1891, I was occupied in considering what would be the best way of utilising the experience acquired during the Franco-Prussian War, so as best to benefit the many wounded. Two spacious houses recently built next to my sanatorium were kindly placed at my disposal, and I now only required the funds necessary for the carrying out of my plan of forming an Ambulance. Fortunately, the funds were not long of being obtained, as a German house with whom I had communicated promised to open a subscription among the German colony at Valparaiso. My plan was simply to form an Ambulance for 50 patients, but on the 29th and 30th (the days following the battle of Placilla), the wounded were brought in in great numbers, and it was impossible to refuse them admission. The Intendente then suggested that I should take also a large building intended for a hotel and adjoining the former houses, promising that the Government should be responsible for all expenses. At the same time several of my colleagues offered their assistance, and the subscriptions amounted to an unexpectedly large sum. Thus the small Ambulance founded by the German colony was converted into a much larger international one." Dr. Von Schrœder then goes on to thank all those who had taken part in the work. Now for my own experiences.

On the day after the battle of Placilla, August 9th, the doctors of Valparaiso and many of the young men rode to the battle-field to render aid to the wounded. On the same morning a gentleman whom I knew asked me to go thither with him, saying that the wounded were in a piteous state, he had been there very early, carrying food and water to the wounded. I had heard of the Ambulance started by Dr. Von Schrœder and thought it best to go there first to ascertain from him where I should be of the most use; he said I could be most useful at the Ambulance, so there I remained, reluctantly abandoning my idea of going to the field of battle. On first going into the hospital I met a scene of indescribable confusion. In the entrance hall were piles of mattresses, blankets, and sheets, iron beds, china ware, and stretchers with poor groaning men arriving continually. The first room I went into was one subsequently used as a dispensary; there were two tables in the room covered with bottles, instruments, &c., and a large box full of bandages, gauze, and lint. In a little room opening off this one and just large enough to hold a table and one chair and with no roof, some doctors were operating, all the other rooms on the ground floor being full of wounded men, laid on mattresses on the floor, only one room being as yet supplied with beds. Workmen were busy, the houses being still unfinished, there was no proper kitchen and the sanitary arrangements were still incomplete.

I was glad to find that one of the surgeons who was operating was a German doctor whom I knew; he had undertaken to attend to all the patients in a large front room, dressing their wounds and performing the most urgent operations. It was truly operating under difficulties in such a small room, without even a table to lay the instruments on, or a piece of mackintosh to put under the patient. Some of the operations performed were: an abdominal section, amputation of foot, resection of part of humerus, extraction of bullets, and many others. By six p.m. it had begun to rain, and the roofless little room had to be abandoned. The patients in bed were easily attended to, but it was another matter in those rooms where they were on mattresses on the floor, with all their clothes still on, and scarcely room to pass between the mattresses. Getting off their clothes was a difficult business, and washing them could not be thought of for the present. We were just going home for the night, about eleven, when the worst case I had yet seen was brought in, a poor fellow with nearly the half of his face shot off, of course, little could be done for him beyond feeding him and putting him in as comfortable a bed as possible. The sufferings of the men on the journey from the battle-field to the hospital must have been terrible; they were brought in coaches devoid of springs, and more shaky than the worst of London "growlers," over twelve miles of road so rough as to defy description. The next two days were like the first, except that plenty of helpers began to arrive, and Dr. Von Schrœder organised the work in some measure, appointing a lady to be over each ward or set of wards, some of the rooms being small, with power to ask as many other ladies to help her as she found necessary. A large room, with windows looking on the Bay, was fitted up as an operation-room, with three tables, case of instruments, basins, &c., and three mozos (hotel waiters) were engaged to take charge of it. To each ward, or rather to each 20 to 30 beds, were assigned a man and a woman as ward assistants, for day duty, and the same for night. During the first week many of the young men of Valparaiso came and made themselves useful, in sitting up at night, or in helping while the doctors were going their rounds. I was placed in charge of a large ward, originally intended to be the dining-room of the hotel, and I also helped to dress the wounds in a group of small wards upstairs; I was fortunate in having the assistance of four ladies, who each came for a certain number of hours in the day, and of three other ladies who between them took the night duty. We had a small room off the large ward fitted up as a kitchen, and there all the small cooking was done; two ladies undertook to act as cooks, and very efficient they were. We were fortunate in getting a wardrobe to keep our linen and dressings in, and also a large table for the middle of the ward; we had also small tables for the men's beds, but nothing to keep their clothes in. The people here are very lazy and dirty, so that it was difficult to keep the ward tidy; the mozo's idea of sweeping was to avoid all the corners, and he had daily to be sent back to sweep under the beds, while the only way to have our patients washed and the ward dusted was to perform these duties ourselves. It was a red-letter day when we got the sailors from an English man-of-war to come and scrub the floors; I hoped the mozo would take a lesson, but he looked on with a bland and child-like smile, and evidently thought the sailors very foolish fellows to work so hard. During the first fortnight the work was very hard, there were so many operations and so much to do to get the work organized, and the patients fairly clean; the slight cases were constantly being drafted off to make way for worse ones.

*(To be continued.)*

### Notes and Queries.

### Queries.

*G.B.*—Will someone kindly tell me which are the best free hospitals for male incurable cases?

### Answers.

*District Nurse.*—Read answer to *Worker*.

*Sister Hilda.*—Thanks the numerous correspondents for their kind replies to her query; they were so many in number that she can only reply to those she thinks suitable.

*Microscope.*—The composition you ask about is called "asphaltum." You can buy it at Newton's in Fleet Street, opposite the Law Courts, in small bottles unless you want a great quantity you had much better buy it than make it up. Brunswick Black does equally well.

*Worker.*—Mrs. Creighton Hale is writing one which will be very excellent; it will be published in three weeks' time; you had better wait for that.

### Nursing Homes.

### V.—CHARING CROSS HOSPITAL.

AFTER once noticing the position which is occupied by this busy little hospital, it is impossible not to dwell on the thought that the site is remarkably convenient as regards the general public's requirements, being, as it were, in a perfect net-work of thoroughfares. At the same time we soon grasp the fact that the building itself, although commodiously designed in all the respects which concern the comforts of the patients, is yet considerably cramped for space which can be devoted to the nurses. The plain exterior of the hospital does not prepare us for the pleasant interior, with the beautifully kept wards and the spotless cleanliness which prevails everywhere; and to the uninitiated this "hospital purity" becomes an ever-new puzzle, whilst even to those who know better the numerous difficulties which wait on its attainment, the eventual and assured success must be a daily encouragement. When the leases of some neighbouring houses fall in, we are encouraged to hope that liberal help will be forthcoming to purchase the property, and to erect on it a complete nurses' home. At present probationers and nurses sleep in sufficiently airy cubicles situated at the top of the building, and they take their meals in a room on the ground floor of the house adjoining the hospital, where are also the apartments occupied by the Matron and Sisters. We are always glad when we find the officials of a hospital both able and willing to arrange for the Sisters, as well as the nurses, to have their bed-rooms distinctly apart from the wards in which their working hours are spent. The different sides of this question are frequently discussed, and we always find that it is one on which two very distinct opinions exist. Many good workers, who are lucky enough to be also good sleepers, maintain that they find no inconvenience from the sounds inseparable from wards, and that they much prefer feeling they are near at hand, whilst to other women who are sensitive to noise, and of less robust constitutions, the constant near vicinity during the hours of night to the sphere of their labours possesses no charm at all, and we incline to think that for all Sisters and charge nurses bed-rooms situated in a different part of the building are desirable wherever attainable.

### Appointments.

[It is requested that successful candidates will send a copy of their applications and testimonials, with date of election, to THE EDITOR, The Lodge, Porchester Square, W.]

ST. SAVIOUR'S INFIRMARY, CHAMPION HILL.—Miss A. L. Crick Burgin, who was trained at Guy's Hospital, and has since held appointments at the Grosvenor Hospital, Bradford Infirmary, and as District Nurse at Newcastle-on-Tyne, has been appointed Night Superintendent of the male wards at this infirmary. Miss Henrietta Whiteford, who was trained at the "London" and was afterwards Sister at the Colonial Hospital, Gibraltar, and Miss Evelyn Boyce, who was trained at the General Hospital, Bristol, have been appointed Sisters.

SANATORIUM, HULL.—Miss Emmeline M. Bann has been appointed Lady Superintendent at this institution. Miss Bann was trained at the Derby Hospital, and subsequently held the post of charge nurse in the scarlet fever wing, making altogether a period of five years spent at Derby; she afterwards held the appointment of Night Superintendent at Gloucester General Infirmary and Eye Institution, and has since been in charge of the two largest blocks at the Royal National Hospital, Ventnor. Miss Bann brings exceptional testimonials and Hull may be congratulated on a wise choice.

### Everybody's Opinion.

*[Correspondence on all subjects is invited, but we cannot in any way be responsible for the opinions expressed by our correspondents. No communications can be entertained if the name and address of the correspondent is not given, or unless one side of the paper only be written on.]*

### NURSES' HOLIDAYS.

"A WOULD-BE TRAMP" writes: Having seen a paragraph in THE HOSPITAL "Nursing Supplement" for April 30th, under the heading of "Everybody's Opinion," I write in response to "A Walking Tour," and quite agree with "Tramp" that a holiday spent in such a way would be most delightful. On one occasion during my hospital training two of us had decided to make such a tour, and were on the eve of making final arrangements, when my chum received an appointment which interfered with her holiday, and our plans were hit on the head. I should like to enter into communication with anybody wishing to be a "tramp" *pro tem.*

"M. V. G. H." writes: In reference to nurses' holidays I should like to tell others of a short one my sister and I took this last March. My sister especially had been working hard all the winter, and was longing for sea breezes. So off we went to Eastbourne, a place new to both of us. We looked out a boarding-house in Bradshaw, so as to save ourselves all bother about meals, &c., and luckily we found we had decided on a very favourite one, where we had to pay 7s. 6d. a day instead of the usual 12s. to 15s. of the summer months. We had fresh breezes, more than enough to satisfy even us; but, with brilliant sunshine, we could sit in the mornings in the terrace-shelters and listen to a good band and watch the sunny waves. We had a capital drive to Beachy Head, and were nearly blown off the summit. Then, for a two-and-sixpenny fare, a four-in-hand went a delightful drive to a meet of the hounds. One evening, at the Devonshire Park Rink, there was a masquerade or carnival, which was a novel and pretty sight to us. Though away less than a week, it seemed a nice long time with so much to amuse and interest us. I find, including the train to and from London, we spent about £2 2s. each, and that included a drive, the carnival, and tea each day at one of the Creameries. We have had delightful holidays in Cornwall and Brittany during our hospital life. I mention Eastbourne to show how well worth while it is to run away to the sea even for a few days, and in winter.

### A HOLIDAY IN SWITZERLAND.

A "NURSE IN YORKSHIRE" writes: In answer to "Tramp," I am hoping to get some of my fellow-workers to join me in my holiday in Switzerland. I was there some time ago, and I suggest this year starting on August 1st, go direct to Paris or Geneva, which ever is most desired, see those two places, then on to Vevey, and the various places of interest, and their name is legion, and either return by the Italian lakes or which ever works out the best. The expedition would have to be done as cheaply as possible, and anyone joining the expedition would be asked only to take a change of dress and linen. We should walk as much as possible, and stay *en route* at any place clean and comfortable. I will forward any particulars to anybody who will like to join for the month's holiday if they will send a stamped envelope.

### Wants and Workers.

[Under this heading, we propose to try whether we can be useful to our readers in making the wants of some known to others who are willing to do what work they can to aid the great cause of curing and cheering the sick. Wants can only be inserted from those who are connected with some institution or association, or who are willing to have their full name and address printed.]

Will any readers of THE HOSPITAL spare a few old clothes, men's, women's, or children's, no matter how worn, for the Deptford Medical Mission. If sent to Nurse Corfmat, at 183, High Street, Deptford, London, S.E., they will be acknowledged in this paper with the Editor's permission.

### Four Months in a Hospital Ward.

### A PERSONAL EXPERIENCE IN A PROVINCIAL HOSPITAL—(*continued*).

### "The Cup which Cheers."

To return to my chronicle of our daily routine: At four o'clock a convalescent patient came round and took from each "locker" two tea-spoonsful of tea; this, when collected, is divided into two portions, one for to-day's tea and the other for to-morrow's breakfast. The tea portion was then put into a large urn and the requisite number of pints of boiling water added thereto. I now proceeded to borrow, as the nurse had suggested, some tea, butter, and sugar. My companions were good fellows every one, for in a minute enough was offered by these poor men to stock a small grocer's shop. A probationer now entered the ward with a great pile of cut bread, and we were soon all busily engaged on "the cup which cheers." I voted this by far the jolliest meal of the day. At about this time two copies of the evening newspapers would be brought up and passed round the ward, a little consideration that shows great thoughtfulness on the part of someone, for this daily glimpse of the outside world helped us on wonderfully. The same clearing of cups and plates and crumbs was done as after dinner, and the rest of the daylight I utilised for the quiet perusal of a book I had brought in with me, varied by an occasional chat with one or other of the "convalescents" who were allowed to "sit up," and from whom I soon learnt the general routine of the ward.

At eight o'clock supper, in the shape of half-a-pint of bread and milk, was served round (sometimes the Matron would give us a special treat at this meal in the shape of a roasted apple), and then the convalescents got into their beds, and by nine o'clock, when the gas was lighted and the night nurse came on duty, everyone had settled down for the night. The night nurse quickly inspected the "bed boards," looked closely at one or two new patients, and then lowered the lights.

I could hear the laboured breathing of the consumptive patient I had seen at the end of the ward when I came in; it sounded all night through like the pumping of an engine in the distance, and I could also hear shocking spasms of coughing at intervals from an old Irishman very ill with bronchitis, and these sounds, together with the weird strangeness of my surroundings, kept me awake the livelong night. I lay and watched the night nurse at the far end of the ward, sitting by the side of the fire and stitching away busily under the subdued light of a little lamp covered with a green shade.

### "Comfortable and Unconcerned."

She seemed comfortable and unconcerned, and you would never think she had the care of seventeen men, some of them ill unto death, but let one of those men moan or make any unusual movement and, in an instant, the shade is turned and a flood of light is thrown upon the bed from whence the sound may have proceeded, and with swift, silent steps this watcher of the night is at his bedside to minister to the suffering patient. So much for the appearance of being "comfortable and unconcerned." Every now and again she would rise and feed the poor dying, consumptive man, and then she would give something to the old Irishman to relieve his coughing, and then she would wake some patient to give a prescribed night dose of medicine; registering all these duties in a report book, returning between each expedition to her stitching, and so on, but all without fuss or hurry. At midnight the house doctor looked in to hear if all was well, and when he had gone the night nurse brewed herself a cup of tea. At about one o'clock the night Superintendent, a sort of Deputy Matron, passed round, and soon afterwards the night nurse brought me a cup of milk, which I drank, and at her suggestion tried hard to go to sleep, but to no purpose; I watched the firelight gleaming along the dark oak boards, and vivid pictures came, unbidden, of the happy days long ago when I was a boy in the dear old house at home. Then my eyes wandered down the ward again to the little figure in black, busily stitching, until I could fancy she was another "Madame Defarge," weaving in her work the destinies of the men under her care.

### "The Dawn of Day."

Now came the dawn of day; how hushed everything seemed, even the dying man breathed more quietly, and the Irishman was asleep at last. The lights were extinguished, the work was folded up, "destinies" and all, and at half-past four o'clock the night nurse was busily engaged getting our breakfasts ready. At a quarter to five o'clock she aroused the convalescents, who promptly dressed, excepting three mischievous boys, who feigned sleep and pretended not to hear the summons. The night nurse was equal to the occasion though, and since they would not hear it, she made them feel it, playfully, of course, but still it had the desired effect, and punctually at five o'clock they were "lending a hand" in carrying round the breakfast portions. Some of the patients had eggs boiled, some eggs beaten up in the tea, some had their bread toasted dry, and others had buttered toast, and all seemed to have some little delicacy in the shape of jam or the like to give a relish to the meal. Breakfast over, a basin of warm or cold water, according to choice, was brought to each patient, about four basins doing duty for the ward, being emptied and refilled by the convalescents as the work proceeded. Now look at the rapidity and precision of the night nurse's movements; the five hardest hours of her duty have commenced, and between now and nine o'clock, "Doctor's parade" I call it, she must not waste a minute if she desires to pass muster when the charge nurse comes to inspect the ward.

### A "Laborious Task."

Beginning at one end, she commences the laborious task of making seventeen beds, on some of which are patients who cannot move, and who have even to be washed, yet she does it all, even to the brushing and combing of their hair. To make the beds of convalescents who have risen is, of course, an easy task, and so is that of those who are strong enough to sit in a blanket on a chair while their bed is made, but the ease and facility with which this little body moved about great, heavy, helpless men, was simply astonishing, and showed the great advantage of training in this as in every other calling in life. Cheerily she pegged away until, at seven o'clock, she was joined by two probationers and the ward maid, and then we presented a busy scene indeed. The floor was first brushed, and it was always a matter of wonder to me where on earth all the "fluff" that was swept up could possibly come from; then it was waxed, and then three or four scrubbers, each about fourteen pounds in weight, affixed to broom handles, were brought to bear upon this polished surface, until to look at the floor was like looking into deep, clear water. All were busy, some scrubbing, some brushing, some washing the tops of the "lockers," some dusting, until, at a quarter to nine o'clock, our industrious little night nurse, now pale and haggard, put the last finishing touches to the coverlets, made a few final entries in the report book, obtained the Night Superintendent's signature thereto, and with her quaint brown teapot and her green lamp under her arm, quietly retired from the stage to make room for the charge nurse. At about half-past eight o'clock the house porter would appear with the letters for the ward, and anxious faces would be lifted from many a pillow in the hope of "news from home"; few correspondents are so well read or so much appreciated as those who send a few cheerful lines to a friend in hospital, for the recipient will know every word by heart before the day is out. A quick walk round to see that everything is trim and square, with here and there a cheery "Are you better this morning?" and here and there a closer look into the faces of those most ill, our gentle charge nurse, having glanced through the night report, and the convalescents having seated themselves each on his own bedside chair, is now ready to receive that monarch of a hospital ward, the house doctor.

(*To be continued.*)

### Darby and Joan.

Darby sat at a paper-strewn table in front of the window, leaning his head on his hands, and pulling nervously at a few white locks with his fingers; whilst silver-haired Joanna, his wife, in a high back chair near the fire-place looked on more or less wonderingly, and turned the heel of a pair of socks that she was knitting without so much as a glance at her work to count and stitch. At length, however, Darby's forefinger and thumb tightened convulsively on the one lock of hair of any respectable length that remained in the wrinkly regions of his forehead, and when they—the aforesaid finger and thumb—had tugged that slippery lock tight out to the end, Darby, their possessor, found speech—addressed vaguely to the ubiquitous god of soliloquy.

"Well now! Well now! If I had only fifty shillings—fifty shillings! T'would do it!"

But Darby was only a retired postman, and his pension could not afford him fifty shillings to complete a wonderful invention which was going to revolutionise the stamping of letters—an invention upon which the old man had been engaged all his life, that he could remember. His wife did not quite understand what the exact process or the presumptive advantages of the invention were to be; but she knew that all her good man's spare time was given up to it, and all his thought except what he expended on her, and as a proper wife she tried to take as much interest in it as he did. She had never had a child or even a hobby to share her heart with her husband.

"Fifty shillings Darby?" she repeated, letting her knitting needles rest a moment. "Why, that's a long sum. When do you want it?"

"I must get it, Joanna; I must get it, or my invention can't go on. It's stuck—tight—and all for fifty shillings."

Now Joanna was in her old age just what she had been in her youth, a very loving and sympathetic woman, and she turned over her husband's words in her mind. "Fifty shillings! Where could she get him fifty shillings?" And though she was ever so loyal to him and his abilities, yet she could not help further querying to herself sceptically, "What would the fifty shillings do for him?" Forty years and more was a long time to be over an invention, and a great many fifty shillings had already gone into the waste-paper basket at her husband's feet. All their savings had evaporated over that invention, but its success came no nearer. Yet, though the old woman must have blushed to assert that she believed in the invention, she did implicitly believe in her husband, and admired him all the more for his pertinacity in the face of failure.

He groaned audibly. It was evidently very apparent to him that he could not gather together fifty shillings to serve his needs. But Joanna's feminine brain set itself to evolving manifold ways and means, and at length came to a concluding happy thought. On the mantel-shelf stood a quaint porcelain vase. It had come into Joanna's possession forty years before—she forgot how; but it was generally valued highly by those who pretended to any knowledge of pottery matters. She wondered if she were to take it to Moses' shop round the corner would he give her money for it. Yet her heart smote her too. She loved that vase. After preserving it proudly all these years, it would go very hard with her to part with it. But though a rebellious tear or two came into her old, dimming eyes, she wiped them quickly with her apron, and resolutely made up her mind.

Going to bed that night Darby ran his fingers along the mantle-shelf in search of a two shilling piece that he had put there to pay the coal man. Doing so, he moved the vase.

"Take care!" his wife cried, almost involuntarily.

"Aye, yes! I'll take care, old woman. I believe now if I broke that bit of china I'd never be the same to you again. You love that, Joanna?"

"Yes, Darby, I love that vase."

"Very much?"

"Yes, very much."

The next afternoon Darby came home from a walk looking terribly discomforted. He had been trying to raise money, but unsuccessfully. Joanna met him with a radiant face. She had been to Moses' bric-a-brac shop, and had taken the vase with her, and had received from the Jew thirty-five shillings for it. To that amount she managed to scrape together enough shillings and pence to bring the total up to the requisite fifty shillings, which sum she now handed over to her husband.

The old man caught his wife round the waist in a fine frenzy of gratitude. He had done that years before with hardly more enthusiasm. He counted the money over, spread it out on the table, made Joanna look at it, touched it, fondled it, and acted just like an old baby, as he was, all the evening. Presently some accident or instinct drew his attention to the mantleshelf. He looked at his wife.

"The vase? Where is the vase?" he asked.

"Gone."

"Gone—no! How? What's become of it?"

Joanna's conscience fluttered a moment, but she replied abruptly—

"Broken!"

"Oh, Joan! My poor Joan." The old man's hand sought his wife's. "How now did it happen?"

Joan wished he would not press her to tell another lie, but she was left no alternative.

"I let it fall this morning, and it broke into pieces," she forced herself to say.

"My poor Joan!" Darby had no words of comfort, but he stroked and caressed her hand sympathisingly.

"It can't be helped, Darby!" she said, with an effort a cheerfulness.

"No, no! But, wife, I'm so sorry—so sorry! And you were so keen on that last night. I'd rather anything had happened."

The news saddened Darby's triumph somewhat; but he started out next day to spend his fifty shillings in the service of his invention. It was nine o'clock when he went out, and Joanna did not expect him back until the evening. She thought to do some washing, and was getting her starch and soda ready when her old husband suddenly burst in on her like a young lover. He carried a brown-paper parcel.

"Open it," he cried eagerly.

Joanna did so. It contained her porcelaine vase.

"Do you like it?" Darby went on breathlessly. "Isn't it like the other one? I wouldn't know the difference, would you?"

"How did you get it?" Joanna asked in a half-dazed way.

Darby hesitated a moment, and reddened even to the roots of his aforementioned frontal lock. "I gave the fifty shillings for it," he stammered out. He was altogether ashamed of himself.

"Where?" Joanna queried.

"At Moses'. I saw it in his window, and thought it so like ——."

The old woman's emotions contended, and her mind despaired; but her heart gave a throb that for the moment went far to shake off the weight of years from her stooping frame, and her memory went back forty years and more, and her thoughts wandered all the way after and back again, and her eyes melted, and, inconsistently enough, withal she felt a queen.

"But the invention, Darby?" she asked tenderly.

There was just a perceptible tremble in Darby's voice. "Oh, that can wait," he answered.

And it is waiting still.

## Figures and Tables

**Figure f1:**